# Case report: Total hip replacement using Innoplant system in a dog with chronic hip luxation and contralateral pelvic limb amputation

**DOI:** 10.3389/fvets.2023.1219617

**Published:** 2023-10-04

**Authors:** William McCartney, Ciprian Ober

**Affiliations:** ^1^NOAH, Dublin, Ireland; ^2^Department of Surgery and Intensive Care, Faculty of Veterinary Medicine, University of Agricultural Sciences and Veterinary Medicine, Cluj-Napoca, Romania

**Keywords:** hip, replacement, dog, amputee, luxation

## Abstract

A 6-year-old female neutered Border Collie presented with an inability to walk. The patient had undergone pelvic limb amputation over a year prior. Orthopedic examination revealed discomfort during hip manipulation, and radiographic examination revealed chronic hip luxation. Total hip replacement was performed using the InnoPlant system, which includes modular screw-in cementless pieces to improve implant stability. Cage rest was for the first four postoperative weeks. Subsequently, assistance was provided while standing until the patient could stand unassisted at 10 postoperative weeks. The clinical and radiological outcomes were excellent 3 months postoperatively. Since it is a new system, there are no data regarding the use of the components of the Innoplant system in dogs with a contralateral amputated pelvic limb. This is the first report describing the use of the Innoplant system for total hip replacement in a dog with a contralateral amputated pelvic limb. Based on the clinical outcomes of this case, the use of an Innoplant prosthesis can be an effective treatment option for dogs with contralateral amputated limbs.

## 1. Introduction

Total hip replacement (THR) is classically performed in dogs with hip dysplasia who develop osteoarthritis (1, 2). Total hip replacement is also indicated for irreparable fractures of the acetabulum or femoral head, failed femoral head and neck excision, and traumatic coxofemoral luxation (acute or chronic) (3). Total hip replacement is usually performed as a salvage procedure for terminal osteoarthritis or medically unresponsive cases; however, the complication rate can be relatively high (3). Several systems are available with various implant configurations (4) using cement or cementless components.

Although acute hip luxation can be treated using various methods (5–8), additional challenges, such as difficulty in repositioning, muscle contraction, re-luxation, and cartilage damage/loss, occur in the case of chronic hip luxation. Thus, the treatment options are limited to femoral head and neck excision or THR (9). Surgical treatment of a three-legged dog has inherent risks, and classic techniques may require modifications (10).

**Figure 1:**
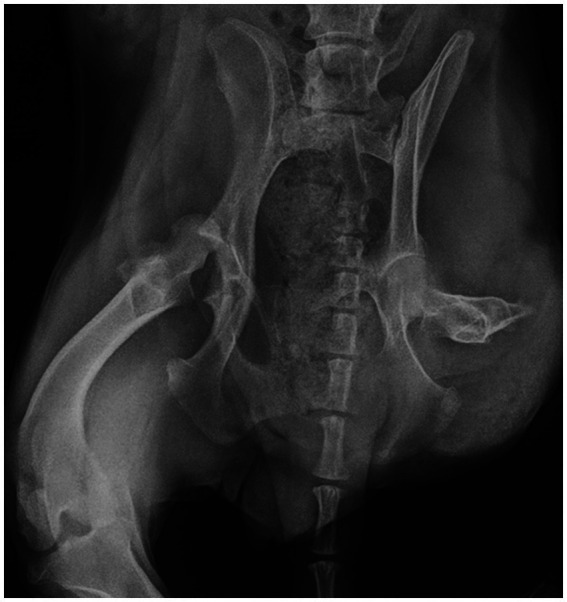
Preoperative radiographs showing craniodorsal hip luxation and contralateral amputated pelvic limb.

This study describes the first case of chronic hip luxation in a dog with a contralateral pelvic limb amputation managed by THR using the InnoPlant system. A 6-year-old female neutered Border Collie weighing 15 kg presented with an inability to walk for more than 2 months. Amputation of one hind leg had been performed over a year before. Orthopedic examination revealed moderate discomfort during the manipulation of the left hip joint. Radiographic examination revealed craniodorsal chronic hip luxation (Figure 1).

## 2. Case description

Conservative treatment consisting of repositioning and Ehmer sling had been attempted unsuccessfully by different veterinarians before referral. Therefore, THR using the InnoPlant system was performed after discussing potential treatment options with the owner.

Medetomidine (0.02 mg/kg IM), synthadon (0.5 mg/kg IM), meloxicam (0.2 mg/kg SC), and cefazolin (22 mg/kg IV) were administered as premedication. Subsequently, anesthesia was induced with propofol (3 mg/kg IV) and maintained with isoflurane (1,000 mg/g). Lateral recumbency was achieved using ties and sandbags to maintain a true lateral position during surgery.

The surgery was performed by a board-certified surgeon (WM). Under aseptic preparation, a craniodorsal approach was applied to the hip. The deep gluteal region was incised using an L-shaped incision, and the femoral head and acetabulum were inspected. Severe cartilage loss from the femoral head was observed intraoperatively. Except for the presence of soft tissue swelling and loose torn tissue, no additional changes were observed in the acetabulum. The trial components of the femoral implants were aligned with the femoral head and neck, and the cementless femoral stem that was deemed the most suitable was selected. The femoral medulla was prepared to accept the stems using appropriately sized reamers. After progressive reaming, the trial stem exhibited a good fit. The femur was then distracted from the acetabular view. An acetabular cup of a suitable size was selected based on the measurements obtained from the pre-operative radiographs. The smallest acetabular reamer was used to initiate acetabular bed formation, and reaming was performed to within 1 mm of the medial aspect of the acetabulum using progressively larger reamers. The depth was confirmed using a depth gage inserted through a small hole drilled in the acetabular center. The edges of the reamed acetabulum were cleared of all soft tissues that could interfere with screw cup engagement. The screw cup was inserted using specific instruments until fully seated at an angle of 45° to the pelvic alignment. Once the cup was securely seated, the femoral stem was inserted into neutral anteversion. During the creation of the bed for the femoral stem, an attempt was made to maintain the femoral stem in the most ventral position without compromising the bone stock to counter the natural tendency of the shortened gluteal muscles to pull the femur into a craniodorsal position. Trial heads of various sizes were used to verify the head size suitable for the femoral stem. Subsequently, the femoral head implant was slid onto the femoral stem, and the hip was reduced. Hip manipulation was performed through all ranges to assess impingement and luxation. All implant interfaces were evaluated for the detection of soft tissue impingement. After routine closure of the muscles, skin, and subcutaneous layers, the dog was placed in a softly bedded kennel for recovery.

Synthadon (0.5 mg/kg, intramuscular) was administered every 8 h for 24 h postoperatively. In addition, meloxicam (0.2 mg/kg, subcutaneous), gabapentin (15 mg/kg orally), paracetamol (10 mg/kg orally), and cephalexin (15 mg/kg, intramuscular) were administered intramuscularly. The dog was discharged the next day. Strict cage rest (1 m × 1 m) was prescribed for 6–8 weeks. Cephalexin (15 mg/kg orally)gabapentin (15 mg/kg orally), and paracetamol (10 mg/kg orally) were administered for 5 days twice per day and meloxicam (0.1 mg /kg orally) was administered for 5 days once per day. Standing was not encouraged during the first 4 postoperative weeks. A gradual increase in assisted standing was recommended after this interval.

A follow-up orthopedic examination at 4 weeks postoperatively revealed that the discomfort during hip manipulation had resolved almost completely. The dog was able to stand unassisted after 10 postoperative weeks. Free walking was gradually increased over the next 6 weeks until the dog was able to walk alone. No minor or major complications were observed during the mid-term clinical and radiographic evaluations.

**Figure 2:**
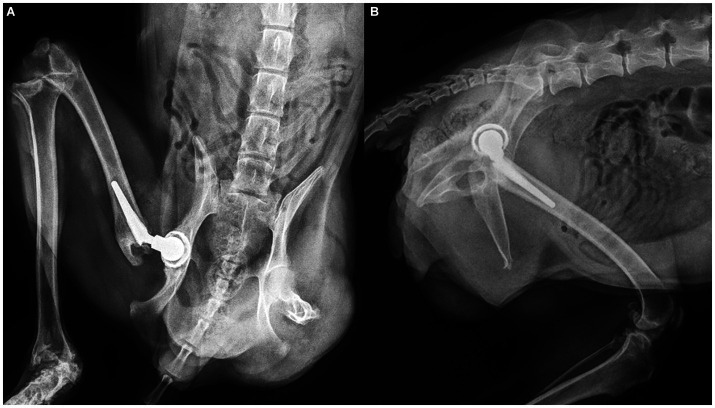
Postoperative radiographs showing the Innoplant total hip replacement system **(A)** ventrodorsal and lateral **(B)** at 12 weeks postoperatively.

The final radiographic examination was performed 12 weeks postoperatively. Radiographs of the hip joint acquired 12 weeks postoperatively revealed no evidence of loosening or changes in implant position, absence of implant-bone interface radiolucency, and no femoral stem subsidence (Figure 2).

Long-term follow-up radiographs were not available; however, the clinical outcomes were assessed as very good by the owner 12 months after THR. The owner assessed his dog’s mobility as very good and perceived that the dog’s quality of life had significantly improved after THR.

## 3. Discussion

This report describes the successful treatment of craniodorsal chronic hip luxation with a contralateral amputated limb via THR using the Innoplant system. To the best of our knowledge, this is the first report to describe the use of the Innoplant system for total hip replacement in a dog with a contralateral amputated pelvic limb.

Luxation of the hip accounts for approximately 90% of all luxations in dogs (11). Chronic luxation is best addressed using femoral head and neck ostectomy or THR (12, 13). Muscle contracture and periarticular fibrosis resulting from chronic hip luxation usually make the insertion of a femoral prosthesis into the acetabular cup challenging (14). Nevertheless, the procedure provided good-to-excellent long-term clinical outcomes in a previous case series (14). All dogs in this study underwent cementless THR (BioMedtrix BFX; BioMedtrix, Whippany, New Jersey).

The Innoplant THR system (INNOPLANT Veterinary, Hannover, Germany) has both cemented and cementless components (15). Cemented (Cemet A Cup) and cementless (Screw Cup) acetabular cups are available, as well as three different femoral stem components: two cementless (HELICA TPS stem and 3Con Stem) and one cemented (Cemet A Stem) (15). The screw-in cementless femoral stem (HELICA TPS stem) and the acetabular (Screw Cup) components are unique to the Innoplant THR system (15). These elements were designed to address some of the concerns with conventional THR systems, especially to increase the stability of the bone-implant interface and decrease micromotion and stress shielding of the bone (16). Additional data on the Innoplant THR system are provided in the paper by Harper (15).

The incidence of contralateral orthopedic diseases after amputation is largely unknown (17), and a standard protocol for orthopedic pathology after amputation has not yet been established. THR is not indicated in cases of contralateral pelvic limb amputation due to the high incidence of postoperative complications, with early luxation of the prosthetic hip as the principal complication (18). Good to excellent long-term clinical outcomes were reported in a previous case series (14) and our patient. Improvements in the designs of new prostheses, such as the Innoplant system, are likely to result in decreased postoperative complications. Gifford et al. (19) reported that the absence of postoperative luxation in their study offers evidence that the current recommendations for THR prosthesis orientation in dogs without contralateral limb amputation are also appropriate for those with amputated contralateral pelvic limbs. The findings of our study also support this result.

Only a few reports in the veterinary literature present the complications associated with the screw-in components of the Innoplant THR system (HELICA Canine Cementless Hip System/HELICA-endoprosthesis) (20–24). Aseptic loosening, resorption of the bone under the collar of the femoral prosthesis, sciatic neuropraxia, femur fissure, and femoral neck fracture are some of the reported complications. No minor or major complications occurred during the recovery period in our patient. The mid-term outcome of the patient was evaluated, and the owner reported “good” to “very good” overall mobility postoperatively. We encouraged progressively assisted standing by the owner without the use of any special devices to prevent excessive loading of the prostheses and soft tissues (19).

The performance of THR in dogs with chronic hip luxation presented obvious surgical difficulties in our case, which is in agreement with those reported in other studies (19). Several factors must be considered during the management of chronically dislocated hip joints, such as the shortened gluteal muscles after the femoral head sitting on the dorsal acetabulum for an extended period. This posed difficulty in reduction and was a major risk factor for postoperative hip re-luxation. To overcome this complication, the femoral stem was inserted more ventrally. We speculated that this minor modification was a significant factor in preventing postoperative prosthetic hip luxation.

The most important limitations of this study were the small sample size and short mid-term radiographic follow-up. The reported follow-up period was insufficient to identify potential long-term complications. Future prospective studies with longer follow-up periods would enable the evaluation of potential late complications, such as aseptic loosening, periprosthetic femoral fractures, or implant failure. Furthermore, this case report was also unable to standardize the perioperative protocol for the Innoplant system.

To the best of our knowledge, there have been no reports on the management of chronic hip luxation in dogs with contralateral amputated pelvic limbs with screw-in endoprostheses. The Innoplant THR system may be a feasible option for THR in dogs with contralateral amputated pelvic limbs.

## Data availability statement

The original contributions presented in the study are included in the article/supplementary material, further inquiries can be directed to the corresponding author.

## Ethics statement

Ethical approval was not required for the studies involving animals in accordance with the local legislation and institutional requirements because ethical review and approval were not required for the animal because this is a case report of examinations and surgery performed for the purpose of patient treatment, and no action contrary to treatment was performed. Written informed consent was obtained from the owners for the participation of their animals in this study.

## Author contributions

WM performed the clinical and surgical management. WM and CO prepared and edited the manuscript. CO contributed to conception of the case report and revised the manuscript. All authors contributed to the article and approved the submitted version.
